# Alpha-fetoprotein and des-gamma-carboxy prothrombin can predict the objective response of patients with hepatocellular carcinoma receiving durvalumab plus tremelimumab therapy

**DOI:** 10.1371/journal.pone.0311084

**Published:** 2024-09-25

**Authors:** Issei Saeki, Shigeo Shimose, Tetsu Tomonari, Takanori Ito, Joji Tani, Yasuto Takeuchi, Naoki Yoshioka, Takehito Naito, Mamiko Takeuchi, Satoru Kakizaki, Takeshi Hatanaka, Kyo Sasaki, Tetsuya Yasunaka, Masahiro Sakata, Hideki Iwamoto, Satoshi Itano, Tomotake Shirono, Norikazu Tanabe, Takafumi Yamamoto, Yuki Kanayama, Atsushi Naganuma, Sohji Nishina, Motoyuki Otsuka, Hideki Kobara, Hiroki Kawashima, Tetsuji Takayama, Takumi Kawaguchi, Takahiro Yamasaki, Taro Takami, Hepatology InVestigator Experts in Japan (HIVE-J) Study Group

**Affiliations:** 1 Department of Gastroenterology and Hepatology, Yamaguchi University Graduate School of Medicine, Yamaguchi, Japan; 2 Division of Gastroenterology, Department of Medicine, Kurume University School of Medicine, Kurume, Japan; 3 Department of Gastroenterology and Oncology, Institute of Biomedical Sciences, Tokushima University Graduate School of Medicine, Tokushima, Japan; 4 Department of Gastroenterology and Hepatology, Nagoya University Graduate School of Medicine, Nagoya, Aichi, Japan; 5 Department of Gastroenterology and Neurology, Kagawa University, Faculty of Medicine, Kagawa, Japan; 6 Department of Regenerative Medicine, Center for Innovative Clinical Medicine, Okayama University Hospital, Okayama, Japan; 7 Department of Gastroenterology and Hepatology, Japanese Red Cross Aichi Medical Center Nagoya Daiichi Hospital, Nagoya, Aichi, Japan; 8 Department of Gastroenterology, Toyohashi Municipal Hospital, Toyohashi, Aichi, Japan; 9 Department of Gastroenterology, Anjo Kosei Hospital, Anjo, Japan; 10 Department of Clinical Research, NHO Takasaki General Medical Center, Takasaki, Gunma, Japan; 11 Department of Gastroenterology, Gunma Saiseikai Maebashi Hospital, Maebashi, Japan; 12 Department of Gastroenterology and Hepatology, Kawasaki Medical School, Kurashiki, Japan; 13 Department of Gastroenterology, Fukuyama City Hospital, Okayama, Japan; 14 Department of Gastroenterology, NHO Fukuyama Medical Center, Fukuyama, Japan; 15 Iwamoto Internal Medical Clinic, Kitakyusyu, Japan; 16 Department of Gastroenterology and Hepatology, Kurume Central Hospital, Kurume, Japan; 17 Division of Laboratory, Yamaguchi University Hospital, Yamaguchi, Japan; 18 Department of Gastroenterology, NHO Takasaki General Medical Center, Takasaki, Gunma, Japan; 19 Department of Gastroenterology and Hepatology, Okayama University Hospital, Okayama, Japan; 20 Department of Oncology and Laboratory Medicine, Yamaguchi University Graduate School of Medicine, Yamaguchi, Japan; Kaohsiung Medical University Hospital, TAIWAN

## Abstract

Durvalumab plus tremelimumab (Durva/Treme) combined immunotherapy is the first-line therapy recommended for unresectable hepatocellular carcinoma (HCC). Since sequential therapy is more effective in improving prognosis, tumor markers have been used as predictive biomarkers for response to systemic therapy. This study aimed to investigate the predictive ability of objective response (OR) by tumor markers for Durva/Treme therapy against HCC. In this multicenter study, 110 patients with HCC who received Durva/Treme therapy were retrospectively enrolled. The OR rate was 15.5%. To aid early decision-making regarding OR, we evaluated the predictors contributing to OR in two steps: before (first step) and 4 weeks after (second step) treatment induction. Changes in tumor markers (alpha-fetoprotein [AFP] and des-gamma-carboxy prothrombin [DCP]) from baseline to 4 weeks after treatment (ΔAFP/ΔDCP) were included as the input factors. In the first step, multivariable analysis identified only the baseline AFP level (odds ratio 3.497, *p* = 0.029) as a predictor of OR. Patients with AFP ≥ 400 ng/mL had a significantly higher OR rate than those with < 400 ng/mL (28.2 vs. 8.5%, *p* = 0.011), and there was no significant difference in progression-free survival (PFS) between the two groups. When AFP/DCP response was defined as a ≥10% reduction from baseline, multivariable analysis showed that AFP response (odds ratio 6.023, *p* = 0.042) and DCP response (odds ratio 11.657, *p* = 0.006) were both independent predictors of OR in the second step. The PFS of patients with AFP or DCP response was significantly longer than that of patients without AFP or DCP response. The study demonstrated that the use of AFP and DCP can predict the OR of patients with HCC receiving Durva/Treme therapy.

## Introduction

Combined immunotherapy is the first-line treatment for unresectable hepatocellular carcinoma (HCC). Following the approval of atezolizumab (anti-programmed cell death ligand -1 [PD-L1] antibody) plus bevacizumab (anti-vascular endothelial growth factor [VEGF] antibody) (Atezo/Bev) [1] in 2020, durvalumab (anti-PD-L1 antibody) plus tremelimumab (anti-cytotoxic T-lymphocyte-associated antigen 4 [CTLA-4] antibody) (Durva/Treme) [2] in 2022 and cammrelizumab (anti-programmed cell death-1 antibody) plus rivoceranib (anti-VEGF-tyrosine kinase inhibitor) [3] in 2023 have been reported to be superior to sorafenib regarding overall survival. Currently, various guidelines have proposed several regimens, including molecular targeted agents (MTA), for second-line therapy and beyond [4, 5]. Since sequential therapy is more effective in improving the prognosis of patients with unresectable HCC [6–8], tumor markers have been used as predictive biomarkers of response in such patients [9–12]. Alpha-fetoprotein (AFP) and des-gamma-carboxy prothrombin (DCP) are classical tumor markers in clinical practice. In particular, AFP has a long history as a biomarker, and its effectiveness in diagnostics, surveillance, and therapeutic monitoring has already been established [13]. In combined immunotherapy, AFP has been reported to be a good monitoring marker for Atezo/Bev therapy [9, 14]. Although DCP has been reported to be a prognostic factor in transarterial therapy [15], it has not yet been reported as a predictor of response to existing MTAs or Atezo/Bev therapy [10–12]. In Durva/Treme therapy, the HIMALAYA trial reported that the objective response (OR) rate was only 20.1%, with a median time to response of 2.17 months [2]. Therefore, identifying patients who might benefit from continued Durva/Treme therapy is important. However, the relationship between tumor markers, such as AFP and DCP, and the radiological OR in such patients remains unclear. In this study, we aimed to address the predictive ability of OR by tumor markers.

## Materials and methods

### Patients

**Fig 1:**
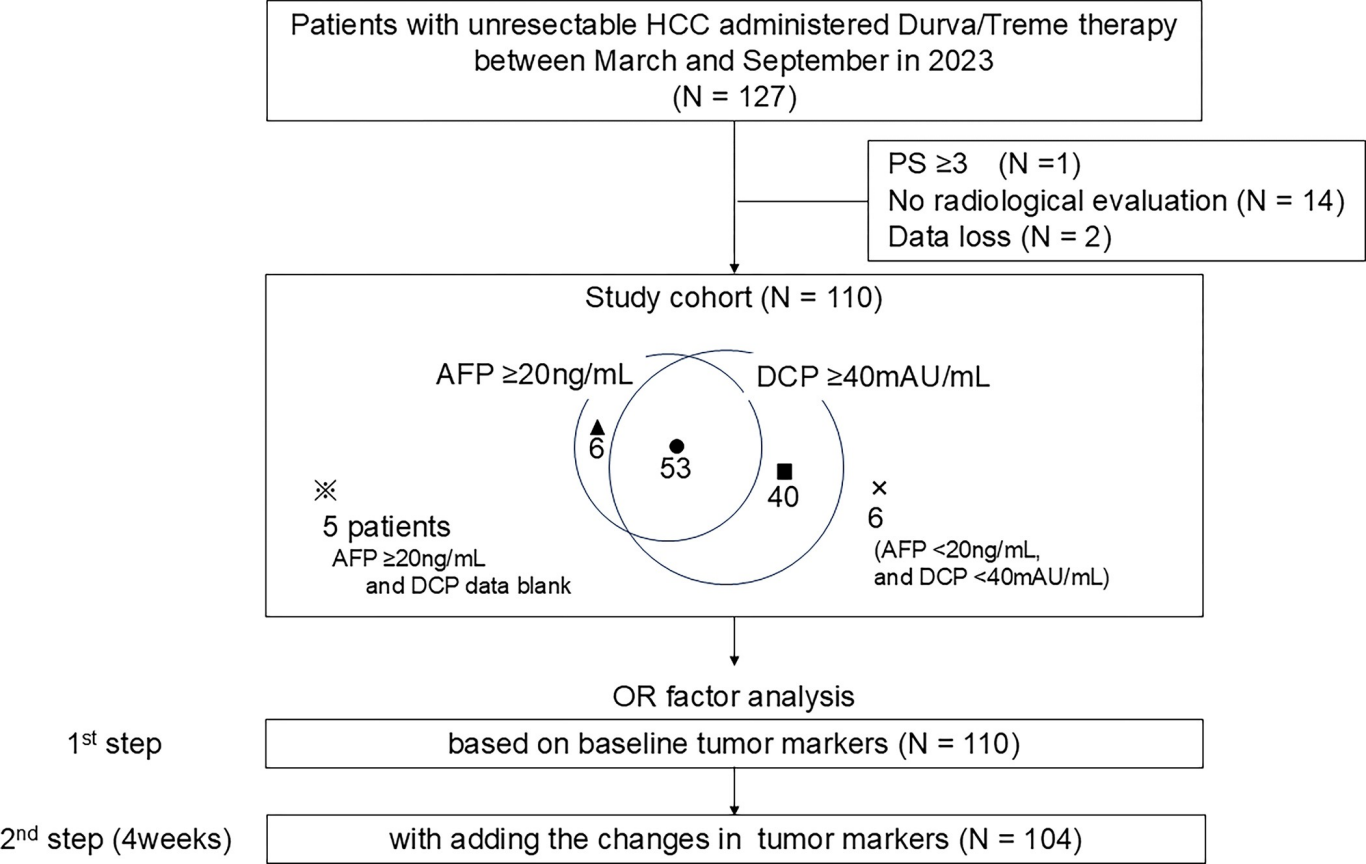
Study chart and positivity of tumor markers at baseline.

This multicenter study retrospectively analyzed existing data of 127 patients with unresectable HCC who received Durva/Treme therapy between March 1, 2023, and September 30, 2023. The involved centers, in Japan, were the following: Kurume University Hospital, Yamaguchi University Hospital, Tokushima University Hospital, Nagoya University Hospital, Kagawa University Hospital, Okayama University Hospital, Japanese Red Cross Aichi Medical Center Nagoya Daiichi Hospital, Toyohashi Municipal Hospital, Anjo Kosei Hospital, National Hospital Organization Takasaki General Medical Center, Gunma Saiseikai Maebashi Hospital, Kawasaki Medical School, Fukuyama City Hospital, and Kurume Central Hospital. The database was accessed after December 7, 2023, when the study was approved by the ethics committee, and all data were collected following anonymization. The inclusion criteria were as follows: AFP and/or DCP had been evaluated as a baseline tumor marker; the patients had undergone at least one followed radiological evaluation for the therapeutic response after the introduction of Durva/Treme therapy; and follow-up of at least 3 months was conducted. The exclusion criteria included performance status (PS) 3 or higher and missing data for the above mentioned variables of interest. Seventeen patients were excluded owing to poor PS (n = 1), lack of radiological evaluation (n = 14), or data loss (n = 2). Finally, 110 patients were included in this study (Fig 1). HCC was diagnosed according to the American Association for the Study of Liver Diseases (AASLD) criteria [4]. The study was conducted in accordance with the guidelines of the Declaration of Helsinki and the protocol was approved by the Institutional Ethics Committee of all institutions. Informed consent for the disclosure of this study was obtained from all patients, with an opt-out option. Further informed consent was waived by the Institutional Ethics Committee (Approval No.: 23153).

### Treatment

Durva/Treme therapy was performed using the same protocol as that of the HIMALAYA trial [2]. Treatment efficacy was evaluated using Response Evaluation Criteria In Solid Tumors ver.1.1 (RECIST v.1.1) [16]. The patients were treated with tremelimumab 300 mg and durvalumab 1500 mg on day 1, followed by durvalumab 1500 mg every 4 weeks. Treatment was continued until progressive disease (PD) or intolerable adverse events (AE) occurred. The initial radiological response was evaluated at 8 weeks, followed by imaging evaluations every 8–12 weeks.

### Assessment of tumor markers

The tumor markers AFP and DCP were measured at baseline (before Durva/Treme therapy) and 4 weeks after treatment induction. The percentage change in tumor marker levels from baseline to 4 weeks after treatment was defined as follows: ΔAFP or ΔDCP = [(AFP or DCP at 4 weeks after treatment induction)—(AFP or DCP at baseline)] / (AFP or DCP at baseline)] × 100.

### Study design

To aid early decision-making for OR, we evaluated predictors contributing to OR at two points: before (first step) and 4 weeks after (second step) treatment induction (Fig 1). We used 400 ng/mL and 1000 mAU/mL as the cutoff values for baseline AFP [17] and DCP [18], respectively. In the first step, we assessed 12 factors in the 110 patients, including age (< 72 or ≥ 72 years), sex (male or female), PS (0 or 1–2), etiology (non-viral or viral), Barcelona Clinic Liver Cancer (BCLC) staging (A–B or C), Child-Pugh class (A or B), up to seven criteria (in or out), macrovascular invasion (absence or presence), extra-hepatic spread (absence or presence), baseline AFP (< 400 or ≥ 400 ng/mL), baseline DCP (< 1000 or ≥ 1000 mAU/mL), and induction line (1^st^ or later). In the second step, in 104 patients with high AFP level (≥ 20 ng/mL) [4] and/or high DCP level (≥ 40 mAU/mL) [19] at baseline, both ΔAFP and ΔDCP were added above the 12 factors.

### Statistical analysis

All continuous variables are expressed as median (interquartile range [IQR]) and compared using the Wilcoxon test. Categorical data are expressed as numbers (%) and compared using Fisher’s exact or chi-squared tests. OR was defined as complete (CR) or partial response (PR), and disease control (DC) as CR or PR or stable disease (SD) in the RECIST v.1.1 evaluation. Factors affecting OR were examined using logistic regression analysis. Explanatory variables were extracted using the stepwise method with a marginal p-value of 0.15 for the selection. For patient outcome, progression-free survival (PFS) was calculated from the induction of Durva/Treme therapy to the confirmation of PD in RECIST v.1.1 or death, and evaluated using a Kaplan–Meier curve, and log-rank test was used for comparison. We conducted a follow-up survey on December 31, 2023, with a median follow-up of 5.9 months. The cutoff for statistical significance was set at a p-value of 0.05. JMP v.16 (SAS Institute, Cary, Vienna, Australia) or StatFlex v.7.0 (Artec Corporation, Osaka, Japan) was used for statistical analysis.

## Results

### Patient characteristics

Patient characteristics are shown in Table 1. The median age was 72 years (IQR, 66–78 years), and 90 (81.8%) patients were male. Regarding liver reserve function, the number of patients with Child-Pugh classes A and B were 100 (90.9%) and 10 (9.1%), respectively. According to the BCLC staging system [5], there were 1 (0.9%), 1 (0.9%), 61 (55.5%), and 47 (42.7%) patients in stages 0, A, B, and C, respectively. Patients with stages 0 and A were both ineligible for local therapy owing to cardiometabolic complications, and Durva/Treme therapy was introduced. AFP and DCP median levels were 54.6 ng/mL and 1360 mAU/mL, respectively. Forty (36.4%) patients were introduced to Durva/Treme therapy as a first-line regimen.

**Table 1:** Patient characteristics.

Characteristic	All patients
**N**	110
**Age**	72 (66–78)
**Sex (male/female)**	90/20
**PS (0/1/2)**	92/16/2
**Body Mass Index (kg/m** ^**2**^ **)**	23.4 (20.9–26.2)
**Cause of HCC (HBV/HCV/Non-B, C)**	21/42/47
**Child-Pugh class A/B**	100/10
**BCLC stage (0/A/B/C)**	1/1/61/47
**Macrovascular invasion (Yes/No)**	19/91
**Extrahepatic spread (Yes/no)**	36/84
**AFP [ng/mL]**	54.6 (4.0–972.3)
**DCP [mAU/mL]**	1360 (151.2–9656.5) *
**Combination therapy (Yes/No)**	9/101^†^
**Treatment line First/Later-line**	40/70
**Follow up duration**	5.9 (4.2–7.5)

* 5 cases blank

^†^ All combination therapies were performed after best-response assessment median (interquartile range)

### Response and survival analysis

**Fig 2:**
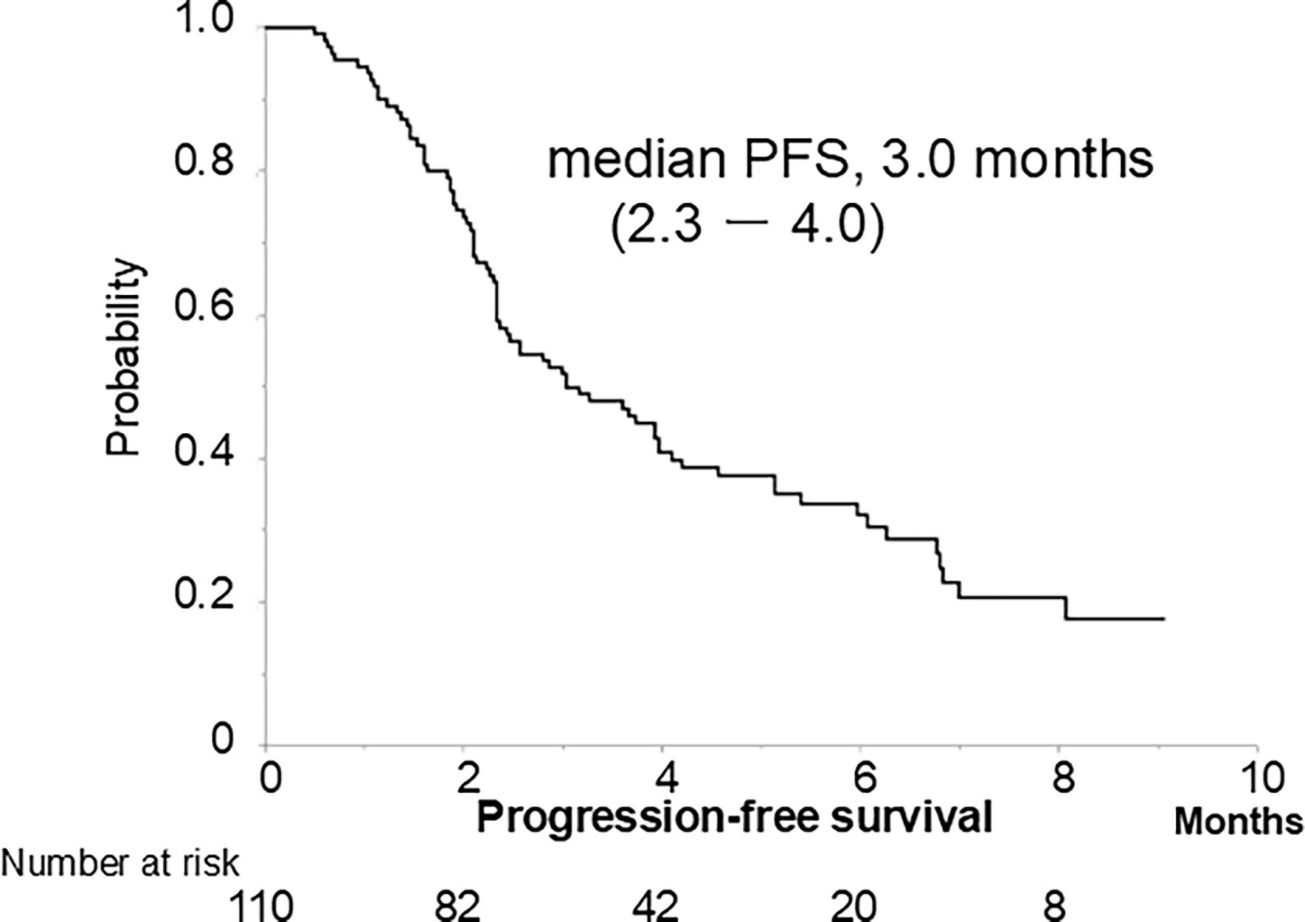
Progression-free survival.

The best treatment responses are presented in Table 2. Three, 14, 42, and 51 patients achieved CR, PR, SD, and PD, respectively, with OR rates of 15.5% and DC rates of 53.6%. Median PFS was 3.0 months (IQR, 2.3–4.0 months) (78 events, 70.9%) (Fig 2).

**Table 2:** Treatment response.

	RECIST ver.1.1	CR	PR	SD	PD	ORR	DCR
		3	14	42	51	15.5%	53.6%
**1**^**st**^ **step**	**Baseline AFP**						
	≥400 ng/mL	2	9	8	20	28.2%	56.3%
	<400 ng/mL	1	5	34	31	8.5%	48.2%
*p* = 0.011 *p* = 0.549							
**2**^**nd**^ **step**	**AFP response**						
	AFP responder	3	7	6	7	66.7%	69.6%
	AFP non-responder	0	5	16	23	33.3%	47.7%
	**DCP response** *p* = 0.005 *p* = 0.122						
	DCP responder	2	7	10	6	36.0%	76.0%
	DCP non-responder	0	4	23	35	6.5%	43.6%

*p* = 0.001 *p* = 0.009

### Tumor markers and their change

**Fig 3:**
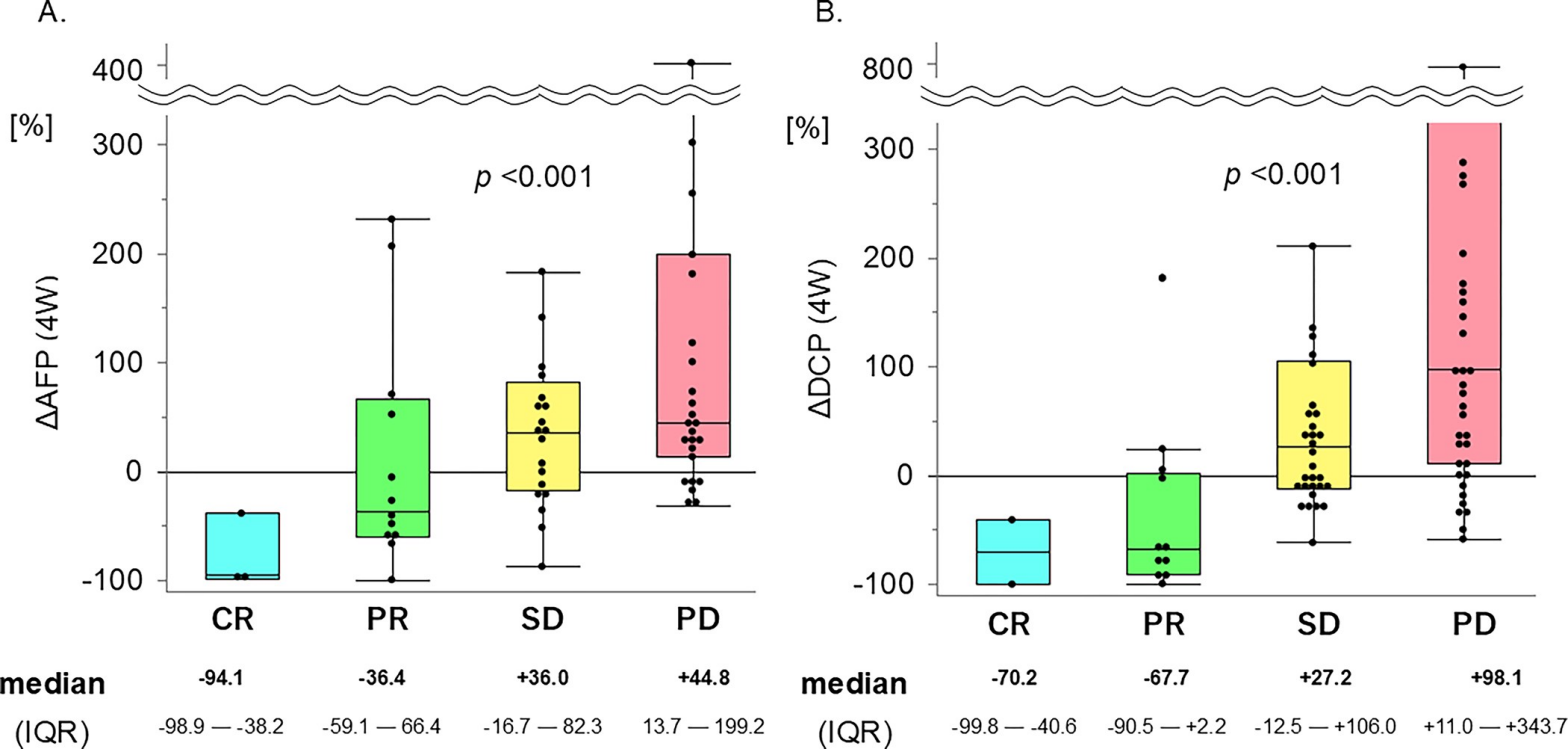
Changes in tumor markers. A. Change in AFP at 4 weeks due to treatment response. Significant reduction in AFP depends on treatment response (N = 62, p < 0.001). B. Change in DCP at 4 weeks due to treatment response. Significant reduction in DCP depends on treatment response (N = 87, p < 0.001).

The patients with high AFP levels (≥ 20 ng/mL) were 64 (58.2%), and those with high DCP levels (≥ 40 mAU/mL) were 93 (84.5%). The distribution of positivity for each marker is shown in Fig 1. DCP was missing in five patients who had a high AFP level (≥ 20 ng/mL). The percentage change is expressed as a water-fall plot (S1A and S1B Fig), and box-and-whisker diagrams (Fig 3A and 3B). According to the best response of the treatment, ΔAFP (N = 62) showed -94.1 / -36.4 / +36.0 / +44.8% (*p* < 0.001), and ΔDCP (N = 87) showed -70.2 / -67.7 / +27.2 / +98.1% (*p* < 0.001), in CR / PR / SD / PD, respectively.

### Objective response predictors

Logistic regression analysis was performed for the OR predictors based on pre-treatment factors. Presence of extra-hepatic spread (odds ratio, 3.424, p = 0.023) and baseline AFP ≥ 400 ng/mL (odds ratio, 4.256, p = 0.009) were significant factors in the univariate analysis; multivariable analysis showed that AFP was the only significant OR predictor (odds ratio, 3.497 [95% CI, 1.140–10.753], *p* = 0.029) (Table 3). Patients with AFP ≥ 400 ng/mL showed a significantly higher OR rate than those with < 400 ng/mL (28.2% vs. 8.5%, *p* = 0.011), whereas DC rate was similar between the two groups (56.3 vs. 48.2%, *p* = 0.549) (Table 2). However, there was no significant difference in PFS between the two groups (S2 Fig).

**Table 3:** Univariate and multivariable analyses for objective response.

		Objective Response		Univariate analysis			Multivariable analysis (1^st^ step)						Multivariable analysis (2^nd^ step)					
				Odds ratio	95%CI	*p-value*	Β	SE(β)	Z	Odds ratio	95% CI	*p-value*	β	SE(β)	Z	Odds ratio	95% CI	*p-value*
2^nd^ step	1^st^ step	Age	<72	0.813	0.288–2.293	0.695												
			≥72	1														
		Sex	male	0.462	0.142–1.503	0.199												
			female	1														
		PS	0	0.390	0.118–1.290	0.123												
			1-	1														
		Etiology	non-viral	1.446	0.493–4.238	0.502												
			viral	1														
		Child-Pugh	A	0.706	0.136–3.653	0.678												
			B	1														
		Up to seven	in	0.463	0.129–1.481	0.184												
			out	1														
		Macrovascular invasion	presence	2.351	0.717–7.713	0.158												
			absence	1														
		Extra-hepatic spread	presence	3.424	1.183–9.907	0.023	0.967	0.566	1.709	3.247	0.853–8.000	0.087	1.308	0.880	1.487	3.704	0.660–20.833	0.126
			absence	1						1						1		
		Baseline AFP	≥400 ng/mL	4.256	1.432–12.644	0.009	1.253	0.572	2.189	3.497	1.140–10.753	0.029						
			<400 ng/mL	1						1								
		Baseline DCP	≥1,000 mAU/mL	1.372	0.451–4.176	0.577												
			<1,000 mAU/mL	1														
		Line	1^st^	1.272	0.443–3.656	0.654												
			later	1														
		AFP response	responder	5.833	1.658–20.522	0.006							1.796	0.882	2.035	6.023	1.068–33.956	0.042
			non-responder	1												1		
		DCP response	responder	8.156	2.220–29.968	0.002							2.456	0.892	2.753	11.657	2.029–66.989	0.006
			non-responder	1												1		

Next, we focused on the change in tumor markers at 4 weeks after treatment induction (ΔAFP and ΔDCP). We defined AFP response and DCP response as a ≥ 10% reduction from baseline. Univariate analysis affecting OR showed 4 factors: extra hepatic spread (*p* = 0.023), baseline AFP (*p* = 0.009), AFP response (*p* = 0.006), and DCP response (*p* = 0.002). Multivariable analysis showed that AFP response (odds ratio, 6.023; [95% CI, 1.068–33.956], *p* = 0.042) and DCP response (odds ratio, 11.657; [95% CI, 2.029–66.989], *p* = 0.006) were both significant independent factors while baseline AFP was not (Table 3).

### Progression-free survival based on each tumor marker response

**Fig 4:**
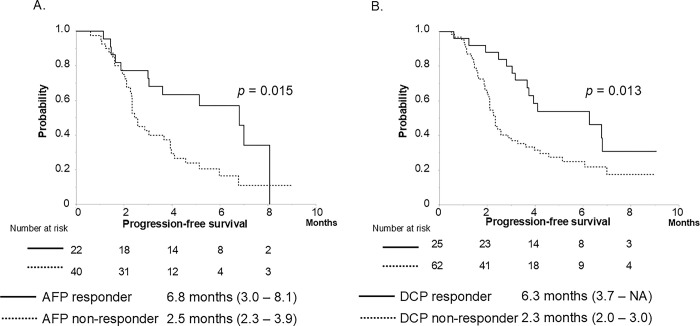
A. Progression-free survival based on AFP response. AFP responders showed significantly longer PFS than non-responders (N = 62, median PFS, 6.8 vs. 2.5 months, *p* = 0.015), B. Progression-free survival based on DCP response. DCP responders showed significantly longer PFS than non-responders (N = 87, median PFS, 6.3 vs. 2.3 months, *p* = 0.013).

The ability to stratify PFS according to the response to each tumor marker was examined. PFS was stratified according to the AFP or DCP response. AFP responders had significantly better PFS than non-responders (median PFS, 6.8 vs. 2.5 months, *p* = 0.015) in 62 patients with AFP ≥ 20 ng/mL (Fig 4A), and DCP responders also showed better PFS than non-responders (median PFS, 6.3 vs. 2.3 months, *p* = 0.013) in 89 patients with DCP ≥ 40 mAU/mL (two patients lacked the DCP data of 4 weeks after treatment induction) (Fig 4B).

## Discussion

In the present study, we demonstrated that AFP and DCP are useful biomarkers for early decision-making regarding OR in patients with HCC who received Durva/Treme therapy. Both AFP and DCP have been used as therapeutic monitoring biomarkers in patients with HCC receiving several treatments, including local therapy, surgical resection, transarterial therapy, and systemic therapy. Currently, many systemic regimens, such as Atezo/Bev, Durva/Treme, and MTAs, are used in clinical practice in many countries. AFP has been reported to predict treatment response to Atezo/Bev [9], sorafenib [20], lenvatinib [10], regorafenib [21], ramucirumab [17], and cabozantinib [22]. However, anti-VEGF agents create a hypoxic environment in the tumor, making it difficult to predict treatment responses to anti-VEGF-related regimens using DCP [23]. Sun et al. reported that AFP and DCP levels could predict the efficacy of anti-PD-1 immunotherapy in patients with HCC [24], although the PD-1 blockade is a heterogeneous monotherapeutic strategy. To the best of our knowledge, this is the first report of Durva/Treme therapy.

In this study, we evaluated the predictors contributing to OR in two steps: before (first step) and 4 weeks after (second step) treatment induction. In the first step, only the baseline AFP level was a predictor of OR. Patients with AFP ≥ 400 ng/mL had a significantly higher OR than those with < 400 ng/mL (28.2 vs. 8.5%, *p* = 0.011). This cutoff value of 400 ng/mL was used in the REACH trials and is considered reasonable. In the HIMALAYA trial, there was no analysis of the response to AFP [2, 25], and our group is the first to show the results using real-world data. Although patients with HCC having baseline AFP ≥ 400 ng/mL have a poor prognosis [26], no significant difference in PFS between patients with AFP ≥ 400 ng/mL and those with AFP < 400 ng/mL was found in this study; this finding may be considered an appositive result. The HIMALAYA trial had shown that patients with AFP ≥ 400 ng/mL contributed to OS at a lower hazard rate than those with AFP < 400 ng/mL (hazard ratio, 0.62 vs. 0.82) [25], hence supporting our PFS results.

In the second step, we focused on the change in tumor markers (ΔAFP and ΔDCP) in patients with high baseline AFP (AFP ≥ 20 ng/mL) and DCP levels (≥ 40 mAU/mL). Since the number of patients with non-viral HCC has been increasing in recent years, the positive rate of AFP is low [27]. In this cohort, owing to the high number of patients with non-viral HCC (47 of 110 patients, 43.7%), 23 patients with non-viral HCC (48.9%) had low AFP levels (< 20 ng/mL). We have shown the distribution of baseline tumor marker levels (*r* = 0.036, *p* = 0.713, S3A Fig). Of the total of 110 patients, 46 (41.8%) had low AFP levels (< 20 ng/mL), although the DCP-positive rate was high (88.6%). Thus, the number of patients with high AFP and/or DCP was 104 (94.5%), which almost dominated the patients in this cohort (Fig 1 and S3A Fig). The radiologic response was significantly associated with ΔAFP or ΔDCP (S1 Fig and Fig 3). We re-examined the correlation between ΔAFP and ΔDCP in 98 patients and measured AFP and DCP at baseline and 4 weeks after treatment induction, regardless of AFP/DCP levels (*r* = 0.060, *p* = 0.555, S3B Fig). Most patients who showed OR were in the responder quadrant (AFP decreased by more than 10% and DCP decreased by more than 10%). Some patients with disease progression were located near the cutoff line (-10% ΔAFP) (red squares), in which only DCP was useful as a monitoring marker. These results suggested that the DCP may complement response prediction in patients with low AFP levels. Interestingly, patients with AFP < 20 ng/mL and DCP < 40 mAU/mL at baseline showed almost no change in either of the tumor markers after 4 weeks despite PD. For such patients, new biomarkers—such as those involving liquid biopsy tests [28]—may need to be considered.

Furthermore, multivariable analysis showed that AFP response (odds ratio 6.023, *p* = 0.042) and DCP response (odds ratio 11.657, *p* = 0.006) were significant predictors of OR (Table 3). Since these predictors are independent, it is important that both tumor markers be used as monitoring biomarkers of treatment response. PFS was stratified according to the AFP or DCP response (Fig 4). We set the cutoff value of AFP/DCP response at a ≥ 10% reduction from baseline to 4 weeks after treatment induction, based on the water-fall plots (S1A and S1B Fig). The sensitivity and specificity of the AFP responses were 67% and 74%, respectively. Using the DCP response, sensitivity and specificity were 78% and 70%, respectively. Based on the above results, we believe that both settings are appropriate.

Therefore, we proposed a “two-step approach for the STRIDE regimen” to predict OR of Durva/Treme therapy early on (S4 Fig). Using baseline AFP ≥ 400 ng/mL, we identified 11 of 17 patients with OR (64.7%) in the first step. Next, using AFP and/or DCP, eight of the 11 patients with OR (72.7%) could be extracted in the second step. In contrast, six of the 71 patients had baseline AFP < 400 ng/mL. Based on positive AFP and/or DCP response, five of six patients with an OR (83.3%) could be extracted in the second step. Finally, using the “two-step approach for the STRIDE regimen,” 13 of 17 patients with OR (76.5%) could be extracted in this cohort. Durva/Treme is a single priming protocol for tremelimumab, with the second and subsequent doses being single-agent durvalumab. If an early OR is not achieved, consideration of transition to next-line therapy may be important to obtain a DC. However, this cohort had a small sample size and a limited number of patients with OR. Therefore, further validation with a larger sample size would be required.

The current study had some limitations. First, the short median follow-up of 5.9 months prevented assessing overall survival. Therefore, the PFS was evaluated in terms of patient outcomes. However, since the median time to progression was 5.4 months in the STRIDE regimen arm [2], PFS might also need to be re-evaluated with further follow-up. On the other hand, the median time to response was 2.17 months (95% CI, 1.84–3.98), suggesting that the treatment response could be adequately evaluated. Second, 9 patients were treated with combination therapies (6 patients underwent transarterial chemoembolization, two underwent radiofrequency ablation, and one underwent hepatectomy during Durva/Treme therapy in this cohort. However, these combination therapies were performed after the evaluation of best response of Durva/Treme therapy; therefore, it cannot be an OR contributor. Although this may have affected PFS, there was no main analysis in this study–an issue that future research may address. Finally, a provisional cutoff value for the AFP/DCP response was used in this study. Several cutoff values have been reported, depending on the regimen [9, 10, 20], and the same regimen may also have different cutoff values [9, 14]. In our cohort, when the cutoff value of AFP/DCP response was set at a ≥ 10% reduction, the specificity of both markers was ≥ 70%. Further validation studies are required to establish the optimal cutoff value for AFP/DCP response.

In conclusion, we demonstrated that AFP and DCP can be used to predict the OR in patients with HCC receiving Durva/Treme therapy.

## Supporting information

S1 Fig **A.** Waterfall plot of change in AFP at 4 weeks. **B.** Waterfall plot of change in DCP at 4 weeks.
(TIF)

S2 Fig Progression-free survival by baseline AFP. There is no difference in progression-free survival between high and low AFP (mPFS, 2.9 vs. 3.6 months, *p* = 0.779).
(TIF)

S3 Fig **A.** Relationship between baseline AFP and DCP. **B.** Relationship between ΔAFP and ΔDCP. RECIST evaluation was identified by color, with CR, PR, SD, and PD indicated in blue, green, orange, and red, respectively. Additionally, the baseline tumor marker values are shown. ● is abnormal for both AFP and DCP, ■ is in the normal range for AFP but abnormal only for DCP, ▲ is abnormal only for AFP and normal range for DCP, × is normal for both AFP and DCP.
(TIF)

S4 Fig Objective response rate according to the two-step approach for the STRIDE regimen. (TIF)
