# Supplementary figures and images for: Alpha-fetoprotein and des-gamma-carboxy prothrombin can predict the objective response of patients with hepatocellular carcinoma receiving durvalumab plus tremelimumab therapy
Source: PLoS One. 2024 Sep 25;19(9):e0311084. doi: 10.1371/journal.pone.0311084 (PMC11423983; doi:10.1371/journal.pone.0311084)

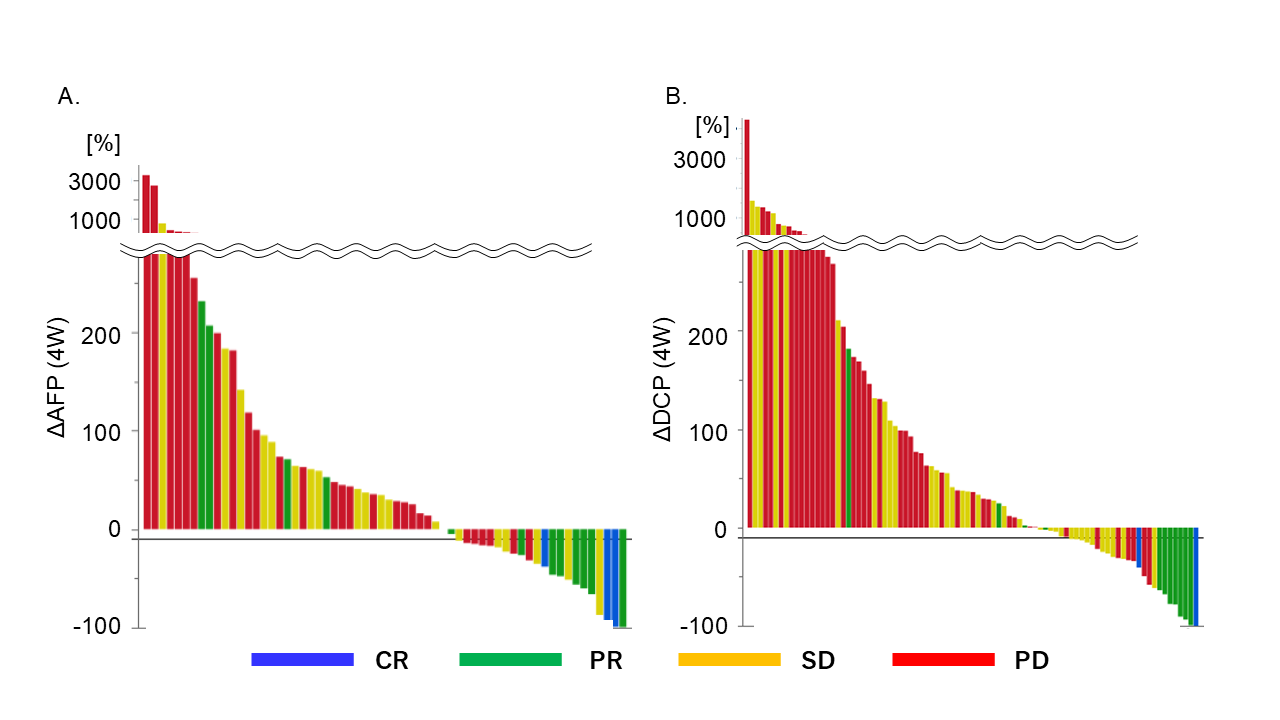

Supplement: S1 Fig — A. Waterfall plot of change in AFP at 4 weeks. B. Waterfall plot of change in DCP at 4 weeks. (TIF) [file pone.0311084.s001.TIF]

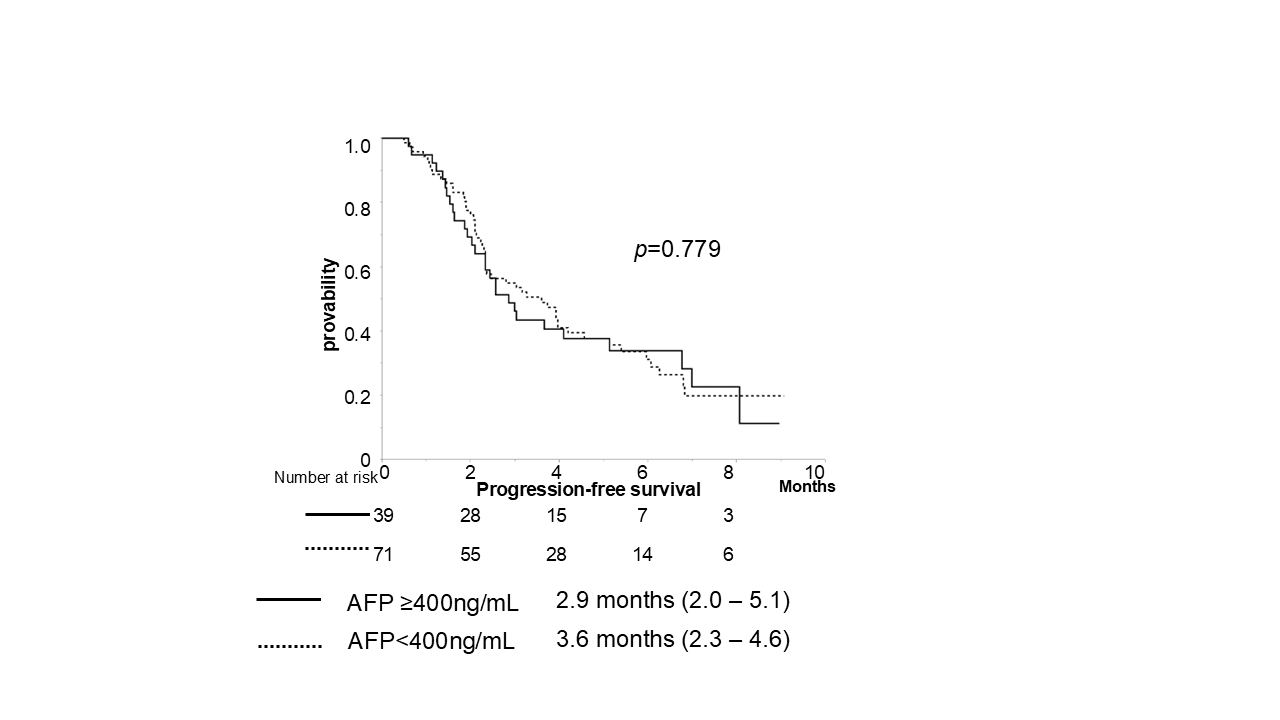

Supplement: S2 Fig — There is no difference in progression-free survival between high and low AFP (mPFS, 2.9 vs. 3.6 months, p = 0.779). (TIF) [file pone.0311084.s002.TIF]

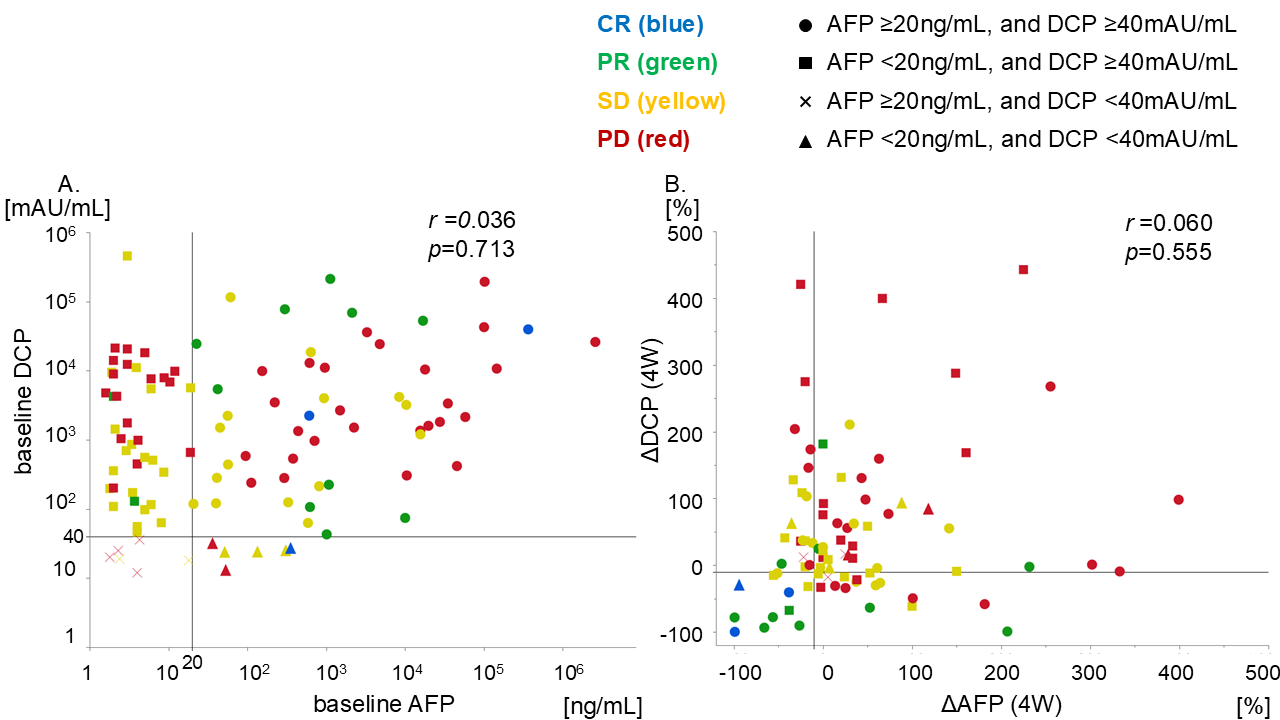

Supplement: S3 Fig — A. Relationship between baseline AFP and DCP. B. Relationship between ΔAFP and ΔDCP. RECIST evaluation was identified by color, with CR, PR, SD, and PD indicated in blue, green, orange, and red, respectively. Additionally, the baseline tumor marker values are shown. ● is abnormal for both AFP and DCP, ■ is in the normal range for AFP but abnormal only for DCP, ▲ is abnormal only for AFP and normal range for DCP, × is normal for both AFP and DCP. (TIF) [file pone.0311084.s003.tif]

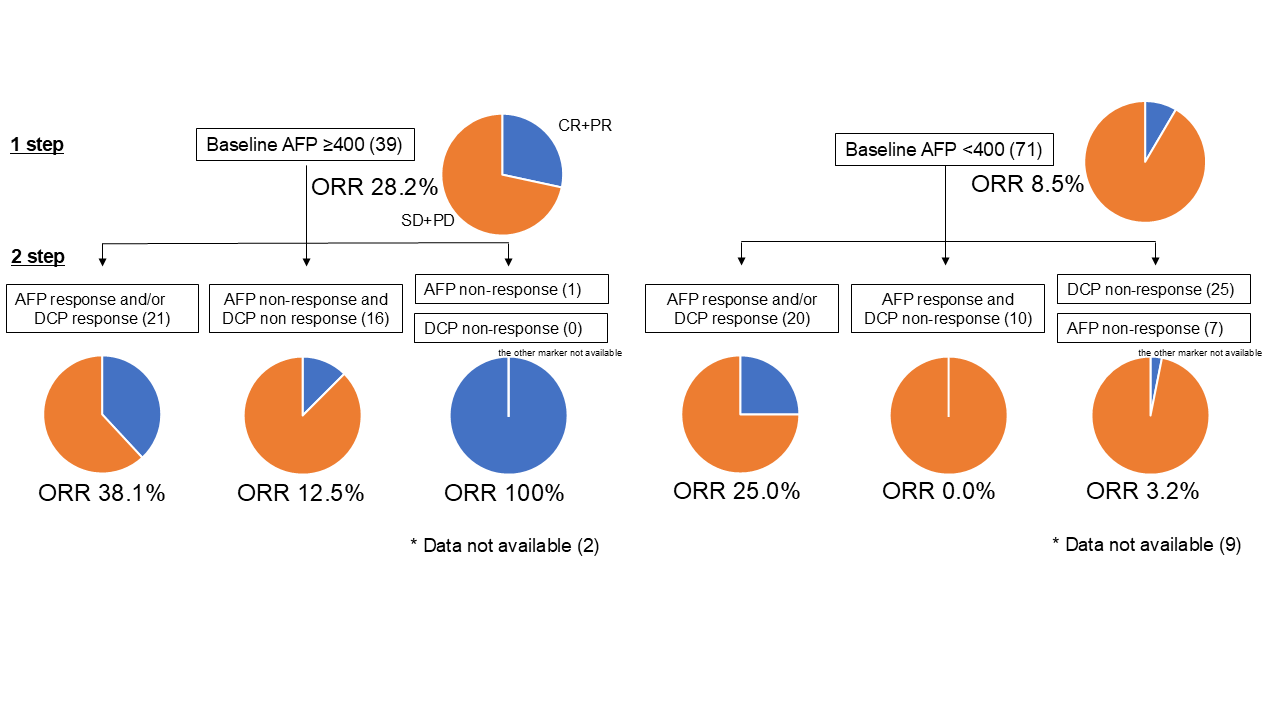

Supplement: S4 Fig — (TIF) [file pone.0311084.s004.TIF]
